# Gamma-glutamyltransferase activity in exosomes as a potential marker for prostate cancer

**DOI:** 10.1186/s12885-017-3301-x

**Published:** 2017-05-05

**Authors:** Kyojiro Kawakami, Yasunori Fujita, Yoko Matsuda, Tomio Arai, Kengo Horie, Koji Kameyama, Taku Kato, Koichi Masunaga, Yutaka Kasuya, Masashi Tanaka, Kosuke Mizutani, Takashi Deguchi, Masafumi Ito

**Affiliations:** 1 Research Team for Mechanism of Aging, Tokyo Metropolitan Institute of Gerontology, 35-2 Sakae-cho, Itabashi-ku, Tokyo, 173-0015 Japan; 2 Department of Pathology, Tokyo Metropolitan Geriatric Hospital, 35-2 Sakae-cho, Itabashi-ku, Tokyo, 173-0015 Japan; 3 Department of Urology, Gifu University Graduate School of Medicine, 1-1 Yanagido, Gifu, Gifu 501-1193 Japan; 4 Department of Urology, Tokyo Metropolitan Geriatric Hospital, 35-2 Sakae-cho, Itabashi-ku, Tokyo, 173-0015 Japan; 5 Department of Clinical Laboratory, Tokyo Metropolitan Geriatric Hospital, 35-2 Sakae-cho, Itabashi-ku, Tokyo, 173-0015 Japan

**Keywords:** Exosome, γ-glutamyltransferase 1, γ-glutamyl transpeptidase, Prostate cancer, Benign prostatic hyperplasia, Diagnostic marker

## Abstract

**Background:**

Exosomes or extracellular vesicles have the potential as a diagnostic marker for various diseases including cancer. In order to identify novel exosomal markers for prostate cancer (PC), we performed proteomic analysis of exosomes isolated from PC cell lines and examined the usefulness of the marker in patients.

**Methods:**

Exosomes isolated by differential centrifugation from the culture medium of androgen-dependent LNCaP prostate cancer cell line and its sublines of partially androgen-independent C4, androgen-independent C4–2 and bone metastatic C4–2B were subjected to iTRAQ-based proteomic analysis. Exosomes were also isolated by immunocapture and separated by size exclusion chromatography and density gradient centrifugation. Protein expression was determined by Western blot analysis. GGT activity was measured using a fluorescent probe, γ-glutamyl hydroxymethyl rhodamine green (gGlu-HMRG). Immunohistochemical analysis of tissues was performed using anti-GGT1 antibody.

**Results:**

Among proteins upregulated in C4–2 and C4–2B cells than in LNCaP cells, we focused on gamma-glutamyltransferase 1 (GGT1), a cell-surface enzyme that regulates the catabolism of extracellular glutathione. The levels of both GGT1 large and small subunits were elevated in exosomes isolated from C4–2 and C4–2B cells by differential centrifugation and by immunocapture with anti-CD9 or -prostate-specific membrane antigen (PSMA) antibody. In cell lysates and exosomes, GGT1 expression correlated with GGT activity. Size exclusion chromatography of human serum demonstrated the presence of GGT activity and GGT1 subunits in fractions positive for CD9. Density gradient centrifugation revealed the co-presence of GGT1 subunits with CD9 in exosomes isolated by differential centrifugation from human serum. Since GGT activity correlated with GGT1 expression in serum exosomes isolated by differential centrifugation, we measured serum exosomal GGT activity in patients. Unexpectedly, we found that serum exosomal GGT activity was significantly higher in PC patients than in benign prostatic hyperplasia (BPH) patients. In support of this finding, immunohistochemical analysis showed increased GGT1 expression in PC tissues compared with BPH tissues.

**Conclusions:**

Our results suggest that serum exosomal GGT activity could be a useful biomarker for PC.

**Electronic supplementary material:**

The online version of this article (doi:10.1186/s12885-017-3301-x) contains supplementary material, which is available to authorized users.

## Background

Exosomes or extracellular vesicles (EV) are microvesicles with a diameter of 40–150 nm that are secreted from various cells [1]. Numerous proteins, miRNAs, RNAs and DNAs are contained in exosomes and their molecular signature largely reflects that of the cells from which they are originated. Exosomes exist in the body fluids such as blood and urine and thus are expected to be a new marker for various diseases including cancer. Yoshioka et al. demonstrated that CD147 embedded in cancer-linked EV in blood can be used for detection of colorectal cancer [2]. Melo et al. recently reported that exosomes expressing glypican-1 in blood can differentiate patients with pancreatic cancer from healthy subjects and those with benign pancreatic disease [3].

Prostate cancer (PC), one of the most common male cancer, is the second-leading cause of cancer death among men in the United States [4]. PC well responds to androgen deprivation therapy, but 10 to 20% of patients develop castration-resistant prostate cancer (CRPC) [5]. In patients with advanced CRPC, bone metastasis is commonly found. Docetaxel, a microtubule-stabilizing taxane, has been used as the first-line chemotherapy for CRPC, but there is a finite amount of time before acquiring resistance [6, 7]. The recent introduction of cabazitaxel, enzalutamide and abiraterone has expanded treatment options for metastatic CRPC patients [8].

Prostate-specific antigen (PSA) has been commonly used as a marker for PC, but it cannot differentiate PC from benign prostatic hyperplasia (BPH) unless PC is advanced and shows much higher serum PSA levels than BPH [9, 10]. In conjunction with measuring PSA levels, imaging modalities such as CT, MRI and bone scan are recommended to monitor the status of patients. There are numerous reports that identified potential markers to diagnose PC, to diagnose progression or aggressiveness of CRPC and to predict prognosis of PC [11]. Since serial prostate biopsy is not usually performed due to its invasiveness and inaccuracy, it would be of great benefit if PC could be diagnosed and monitored by exosomes in the body fluids. We and others have demonstrated that prostate-specific membrane antigen (PSMA) and P-glycoprotein (P-gp) encoded by multi-drug resistance protein 1 (MDR1) expressed on the surface of blood exosomes could be a marker for PC and taxane-resistant CRPC, respectively [12–15]. We have also recently reported the potential of integrin β4 and vinculin in exosomes as markers for progression and aggressiveness of CRPC [16].

In the present study, we aimed to identify novel exosomal markers for PC especially those for castration-resistance and bone metastasis by analyzing exosomes secreted from PC cell lines including androgen-dependent LNCaP cell line and its sublines of partially androgen-independent C4, androgen-independent C4–2 and bone metastatic C4–2B [17, 18]. Among proteins identified by proteomic analysis, we focused on gamma-glutamyltransferase 1 (GGT1), a cell-surface enzyme that regulates the catabolism of extracellular L-gamma-glutamyl-L-cysteinylglycine (glutathione; GSH). Since GGT activity correlated with GGT1 expression in serum exosomes isolated by differential centrifugation, we measured GGT activity in patients. Contrary to our expectation, we found that serum exosomal GGT activity was significantly higher in PC patients than in BPH patients, which was supported by the finding that GGT1 expression was increased in PC tissues compared with BPH tissues. Altogether, we have identified serum exosomal GGT activity as a novel marker to diagnose PC or to distinguish PC from BPH.

## Methods

### Cell culture

Human prostate cancer LNCaP cell line and its sublines of C4, C4–2 and C4–2B cell lines were obtained from the MD Anderson Cancer Center (Houston, TX, USA) and cultured in DMEM/Ham’s F12 (4:1) medium supplemented with 10% fetal bovine serum, 5 μg/mL insulin, 13.65 pg/mL triiodo-thyronine, 4.4 μg/mL apo-transferrin, 0.244 μg/mL d-biotin and 12.5 μg/mL adenine in a humidified atmosphere containing 5% CO_2_.

### Isolation of exosomes by differential centrifugation

Cells (3.5 × 10^6^) seeded on 150-mm dish were cultured for 72 h in DMEM/Ham’s F12 (4:1) medium containing 10% exosome-deprived fetal bovine serum and other supplements described above. Exosomes were isolated from the conditioned medium as previously described [19]. Briefly, the medium was centrifuged at 2000 xg for 10 min to eliminate cells. Second, the supernatant was centrifuged at 12000 xg for 30 min to remove debris. Third, the supernatant was filtered through 0.22 μm polyvinylidene difluoride (PVDF) filter. Finally, exosomes were pelleted by ultracentrifugation at 110,000 xg for 70 min, resuspended in PBS and stored at −80 °C until use.

### Isolation of exosomes by immunocapture

Mouse monoclonal anti-CD9 antibody (BioLegend, San Diego, CA, USA) and anti-PSMA antibody (MBL, Nagoya, Japan) were conjugated with Dynabeads M-270 epoxy magnetic beads (Life Technologies, Eugene, OR, USA) according to the manufacturer’s protocol. The conditioned medium was centrifuged at 2000 xg for 10 min and the supernatant was centrifuged at 12000 xg for 30 min. The supernatant was filtered through 0.22 μm PVDF filter and 30 mL of the filtrate were incubated with 1 mg of the antibody-conjugated beads at 4 °C for 90 min with rotation. The beads were washed 3 times with PBS and resuspended in sample buffer. After separation from magnetic beads, samples were boiled and stored at −20 °C until use.

### Isolation of exosomes by size exclusion chromatography

A commercially available size exclusion chromatography column, EVSecond (GL Science, Tokyo, Japan), was used for isolation of exosomes. After washing with PBS, 500 μL serum was loaded onto the column and eluted with PBS. The first 1 mL of eluate was discarded and thereafter the eluate was collected in 24 fractions of 0.1 mL each.

### Quantitative proteomic analysis

Proteomic analysis was performed as previously described [16]. In brief, exosomes were labeled with iTRAQ reagents using the iTRAQ multiplex kit (AB Sciex, Foster City, CA, USA). Labeled samples were separated and automatically spotted onto a MALDI plate using the direct nanoLC and MALDI fraction system DiNa-MaP (KYA Technologies, Tokyo Japan). Mass spectra were acquired using the AB Sciex TOF/TOF 5800 system operated on the TOF/TOF Series Explorer software version 4.1 (AB Sciex). All MS/MS data were submitted to the ProteinPilot software version 4.5 (AB Sciex). Protein identification was considered to be correct based on the following selection criteria: protein having at least 2 peptides with an ion score above 95% confidence; and protein with protein score (ProtScore) > 1.3 (unused, *p* < 0.05, 95% confidence).

### Western blot analysis

Whole cell lysates were prepared in ice-cold lysis buffer (1% Igepal CA-630, 1% sodium deoxycholate, 0.1% SDS, 150 mM NaCl, 25 mM Tris-HCl [pH 7.6]) containing protease inhibitor cocktail. Cell lysates and exosomes were subjected to electrophoresis on SDS-polyacrylamide gels and transferred to PVDF membranes. After blocking in 5% skim milk, membranes were hybridized with a primary antibody and then with a horseradish peroxidase-linked secondary antibody. After washing, bound proteins were visualized using the ECL Prime Western blotting detection system (GE Healthcare, Little Chalfont, UK) or Immunostar LD (Wako Pure Chemical Industries, Osaka, Japan). Anti-CD9, -PSMA and -β-actin antibodies were obtained from Cell Signaling Technology (Danvers, MA, USA). Antibodies recognizing GGT1 small subunit was purchased from Abnova (Taipei, Taiwan). Anti-GGT1 large subunit and -Alix antibody were from Santa Cruz Biotechnology (Santa Cruz, CA, USA).

### Measurement of CD9 level

The CD9 level in exosomes was determined by a sandwich ELISA. A MaxiSorp micro titer plate (Thermo Fisher Scientific, MA, USA) was coated with 5 μg/mL anti-CD9 antibody (Ancell Corporation, Bayport, MN, USA) in carbonate buffer (pH 9.6) at 4 °C overnight. After washing 3 times with PBS, 200 μL of 1% BSA/PBS was added and incubated at room temperature for 1 h with shaking. After washing, sample was added in a final volume of 100 μL and incubated at room temperature for 2 h. After washing, 0.5 μg/mL biotinylated anti-CD9 antibody (Ancell Corporation) in 1% BSA/PBS was added in a final volume of 100 μL and incubated at room temperature for 1 h. After washing, 1:5000 diluted streptavidin-AP (Roche, Basel, Switzerland) in 1% BSA/PBS was added in a final volume of 100 μL and incubated at room temperature for 1 h. After washing 6 times with PBS, CDP-Star substrate with Emerald II Enhancer (Thermo Fisher Scientific) was added and chemiluminescence was recorded by the EnVision Multilabel Reader (PerkinElmer, MA, USA).

### Measurement of GGT activity

GGT activity was measured using a fluorescent probe, γ-glutamyl hydroxymethyl rhodamine green (gGlu-HMRG), which is commercially called ProteoGREEN-gGlu (Goryo Chemical, Hokkaido, Japan) [20]. Twenty microliter of sample was reacted with 180 μL of 1.11 μM ProteoGREEN-gGlu in PBS in each well of 96-well black plates (Corning, NY, USA). The plate was incubated at room temperature for 1 h and fluorescence intensity (Ex/Em 490/520 nm) was measured using the EnVision Multilabel Reader (PerkinElmer).

### OptiPrep density gradient centrifugation

Five hundred microliter of serum was centrifuged at 12000 xg for 30 min and the supernatant was filtered through 0.22 μm PVDF filter. The filtered sample was diluted with 11 mL of PBS and centrifuged at 110,000 xg for 70 min. The pellet was resuspended in 500 μL of PBS. A stock solution of OptiPrep (60% *w*/*v* iodixanol) (Axis-Shield, Dundee, Scotland) was diluted with 0.25 M sucrose, 10 mM Tris-HCl (pH 7.6) to generate 40%, 20%, 10% and 5% *w*/*v* iodixanol solutions. A discontinuous density gradient was generated by sequential layering of 3 mL each of 40, 20 and 10% (*w*/*v*) iodixanol solutions, followed by 2.5 mL of 5% iodixanol solution in ultracentrifuge tubes. Sample was overlaid on the discontinuous iodixanol gradient followed by centrifugation at 110,000 xg for 16 h. One milliliter fractions were collected from the top of the gradient. Each sample was diluted with 11 mL of PBS and centrifuged at 110,000 xg for 70 min. The pellet was resuspended in PBS and stored at 4 °C until use.

### Collection of blood from patients and isolation of exosomes

This study was approved by the Bioethics Committees of Gifu University and Tokyo Metropolitan Institute of Gerontology and a written informed consent was obtained from all patients. Thirty-nine patients suspicious of PC due to either abnormal MRI findings or elevated PSA levels were recruited. Blood was corrected from patients prior to biopsy. After biopsy, 31 patients and 8 patients were pathologically diagnosed as PC and BPH, respectively. Serum was separated from whole blood by centrifugation at 1800 xg and stored at −80 °C until use. For exosome isolation, 210 μL of serum was centrifuged at 12000 xg for 30 min and the supernatant was filtered through 0.22 μm PVDF filter. The 200 μL of filtered sample diluted with 800 μL of PBS was centrifuged at 100,000 xg for 75 min. The pellet was washed in PBS and centrifuged at 100,000 xg for 75 min. The final pellet was resuspended in PBS and stored at 4 °C until use.

### Immunohistochemical analysis of GGT1

This study was approved by the Bioethics Committees of Tokyo Metropolitan Institute of Gerontology. Formalin-fixed paraffin-embedded biopsies and surgically resected tissue specimens from PC (*n* = 50) and BPH (*n* = 50) patients were stained for GGT1. The tissue sections (3 μm) were subjected to immunostaining using anti-GGT1 antibody raised against the small subunit (Abnova). After deparaffinization, the sections were preheated in Heat processor solution (pH 6.0, Nichirei, Tokyo, Japan) at 100 °C for 30 min. The sections were then incubated with the anti-GGT1 antibody (1:800 in dilution) at 4 °C overnight. Bound antibodies were detected with the Envision kit (Dako Denmark A/S, Glostrup, Denmark) using diaminobenzidine tetrahydrochloride as a substrate. The sections were then counterstained with Mayer’s hematoxylin. Negative control tissue sections were prepared by omitting the primary antibody. In order to evaluate GGT1 expression, the intensity (1, 0+; 2, 1+; 3, 2+; 4, 3+) and percentage (1, 0–25%; 2, 26–50%; 3, 51–75%; 4, 76–100%) of membranous and cytoplasmic GGT1 staining were scored. GGT1 expression in the prostatic glands and prostatic cancer cells was evaluated under ×200 magnification. The score of intensity multiplied by that of percentage was used as the final score for GGT1 expression. Two independent pathologists blinded to the clinical and pathological information performed scoring.

### Statistical analysis

Statistical differences were determined by one-way ANOVA with Tukey’s multiple comparison tests (for comparison among cell lysates and exosomes isolated from cultured cells), Welch’s t**-**test (for comparison among serum PSA concentration, serum GGT activity and serum exosomal GGT activity), Brunner-Munzel test (for comparison between BPH and PC in immunohistochemical analysis) or paired Student’s t**-**test (for comparison between cancerous and non-cancerous lesions in immunohistochemical analysis). Spearman’s rank correlation coefficient was used to evaluate the correlation between GGT activity and GGT1 expression. *p* < 0.05 was considered statistically significant.

## Results

### Identification of GGT1 as a potential exosomal marker for PC based on proteomic analysis of exosomes isolated from PC cells by differential centrifugation

We analyzed androgen-dependent LNCaP cell line and its sublines of C4, C4–2 and C4–2B cell lines [17, 18]. The C4 cell was established from LNCaP cell transplantation under castration and showed low sensitivity to androgen. The C4–2 cell was established from C4 cell transplantation under long term castrated condition and showed androgen independent growth response. The C4–2B cell was established from bone metastasis of C4–2 cell transplantation under castration. Exosomes were isolated from the cell culture medium by differential centrifugation. In order to identify differentially expressed proteins in exosomes, we performed iTRAQ-based quantitative proteomic analysis of exosomes. A total of 153 proteins were detected (Additional file 1: Table S1) and eight proteins were found to be upregulated by more than 1.5-fold in exosomes isolated from C4–2B cells compared with those from parental LNCaP cells (Table 1). Among them was GGT1, a cell-surface enzyme that cleaves extracellular GSH and provides cells with amino acids, thereby increasing the intracellular GSH level [21]. GGT1 was upregulated in C4–2 cells (1.56-fold) as well as in C4–2B cells (1.63-fold). Since serum GGT activity is reported to be elevated in patients with certain types of cancer [22], we focused on GGT1 as a potential exosomal marker for PC in the subsequent studies.

**Table 1:** Proteins upregulated (>1.5 fold) in C4–2B exosomes compared with LNCaP exosomes

Accession number	Protein name	Gene symbol	C4/LNCaP	C4–2/LNCaP	C4–2B/LNCaP
P05106	Integrin beta-3	ITGB3	0.78	1.50	2.00
O60716	Catenin delta-1	CTNND1	1.10	1.58	1.94
P35221	Catenin alpha-1	CTNNA1	0.97	1.51	1.77
P02765	Alpha-2-HS-glycoprotein	AHSG	0.96	1.19	1.71
P19440	Gamma-glutamyltranspeptidase 1	GGT1	0.82	1.56	1.63
P61224	Ras-related protein Rap-1b	RAP1B	0.75	1.25	1.58
P62258	14–3-3 protein epsilon	YWHAE	1.66	1.29	1.54
P53985	Monocarboxylate transporter 1	SLC16A1	1.13	1.56	1.51

### Upregulation of GGT1 expression in exosomes isolated from C4–2 and C4–2B cells by differential centrifugation

**Fig. 1:**
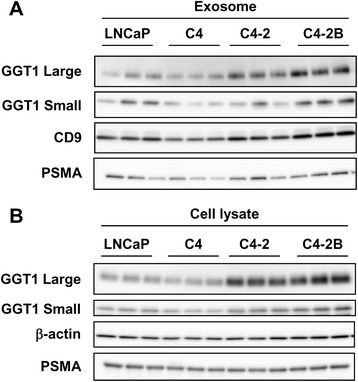
GGT1 expression in exosomes isolated from PC cells by differential centrifugation. Exosomes were isolated from the culture medium of LNCaP, C4, C4–2 and C4–2B cells by differential centrifugation. Exosomes (**a**) and cell lysates (**b**) were subjected to Western blot analysis for GGT1 large and small subunits, CD9, PSMA and β-actin

GGT1 is comprised of a large subunit that anchors the enzyme to the cell membrane and a small subunit that binds to and catalyzes the first step in the degradation of extracellular GSH [23]. Western blot analysis showed elevation of GGT1 large and small subunits in exosomes isolated from C4–2 and C4–2B cells compared with LNCaP and C4 cells (Fig. 1a). PSMA as well as CD9, an exosomal marker, were detected in exosomes isolated from four cell lines. Consistent with increased expression in exosomes, both GGT1 large and small subunits were upregulated in C4–2 and C4–2B cells, whereas the PSMA expression level was similar among 4 cell lines (Fig. 1b). These results confirmed that GGT1 expression in exosomes was increased in castration-resistant C4–2 and bone metastatic C4–2B cell lines.

### Upregulation of GGT1 expression in exosomes isolated from C4–2 and C4–2B cells by immunocapture

**Fig. 2:**
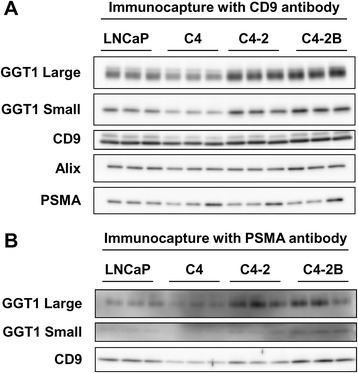
GGT1 expression in exosomes isolated from PC cells by immunocapture. The culture medium of LNCaP, C4, C4–2 and C4–2B cells (30 mL) were incubated with magnetic beads (1 mg) conjugated with anti-CD9 (**a**) or -PSMA (**b**) antibody at 4 °C for 90 min. Whole immunocaptured exosomes were subjected to Western blot analysis for GGT1 large and small subunits, CD9, Alix and PSMA

When exosomes were isolated from the cell culture medium by the immunocapture method using anti-CD9 antibody, the levels of GGT1 large and small subunits were elevated in C4–2 and C4–2B cells, while expression of PSMA as well as exosomal markers, CD9 and Alix, showed no major difference among cell lines (Fig. 2a). Similarly, GGT1 large and small subunits were upregulated in exosomes captured from C4–2 and C4–2B cells by anti-PSMA antibody (Fig. 2b). These results suggested that distinct subsets of exosomes positive for CD9 or PSMA exhibited increased expression of GGT1.

### Correlation of GGT activity with GGT1 expression in exosomes isolated from PC cells

**Fig. 3:**
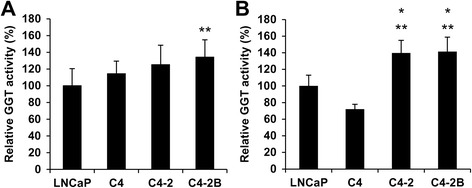
GGT activity in cell lysates and exosomes isolated from PC cells by differential centrifugation. Cell lysates (**a**) and exosomes isolated from the culture medium of LNCaP, C4, C4–2 and C4–2B cells by differential centrifugation (**b**) were mixed with gGlu-HMRG. After incubation at room temperature for 1 h, fluorescence intensity (Ex/Em 490/520 nm) was measured by using microplate reader. GGT activity is shown as a percentage of LNCaP cells. *p < 0.05, compared with LNCaP cells, **p < 0.01, compared with C4 cells

Here we measured GGT activity in exosomes using a fluorescence imaging probe, γ-glutamyl hydroxymethyl rhodamine green (gGlu-HMRG) that is activated by cleavage of glutamate with GGT [20]. GGT activity was measurable in both cell lysates and exosomes isolated from LNCaP, C4, C4–2 and C4–2B cells. There was a significant increase in GGT activity in C4–2B cells compared with LNCaP cells (Fig. 3a). On the other hand, GGT activities were higher in exosomes isolated from C4–2 and C4–2B cells than in those from LNCaP and C4 cells (Fig. 3b). More importantly, GGT activity in exosomes correlated with the expression levels of GGT1 large and small subunits in exosomes among 4 cell lines (Fig. 1a). These results indicated that exosomal GGT activity could be used as an alternative to exosomal GGT1 expression.

### GGT activity and GGT1 expression in exosomes isolated from human serum

**Fig. 4:**
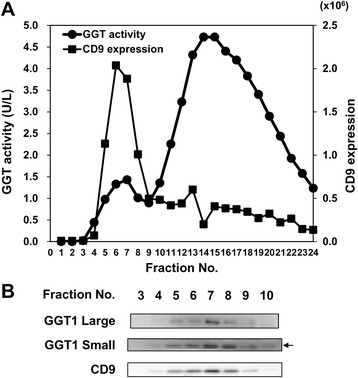
GGT activity and GGT1 expression in exosomes isolated from human serum by SEC. Serum of a healthy individual (500 μL) was subjected to SEC. **a** CD9 expression in each fraction was measured by a sandwich ELISA. GGT activity in each fraction was determined by incubation with gGlu-HMRG at room temperature for 1 h and measurement of fluorescence intensity (Ex/Em 490/520 nm) using microplate reader. **b** The fractions 3–10 collected from a healthy individual were subjected to Western blot analysis for GGT1 large and small subunits and CD9. The *upper band* of the doublet corresponds to the GGT1 small subunit (shown by *arrow*)

**Fig. 5:**
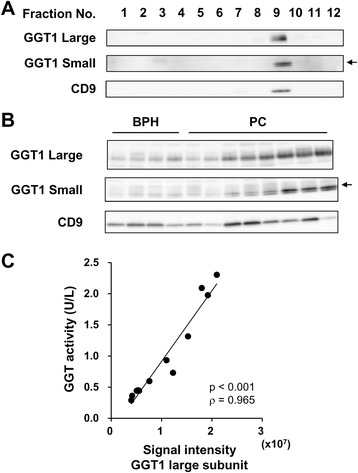
GGT1 activity and GGT1 expression in exosomes isolated from human serum by differential centrifugation. **a** Exosomes isolated from serum of a healthy individual (500 μL) by differential centrifugation were separated by OptiPrep density gradient centrifugation. After ultracentrifugation, fractions were subjected to Western blot analysis for GGT1 large and small subunits and CD9. The *upper band* of the doublet corresponds to the GGT1 small subunit (shown by *arrow*). **b** Serum exosomes isolated by differential centrifugation from BPH (*n* = 4) and PC (*n* = 8) patients were subjected to Western blot analysis for GGT1 large and small subunits and CD9 as well as measurement of GGT activity using gGlu-HMRG. **c** Spearman’s rank correlation coefficient analysis was performed between the signal intensity of GGT1 large subunit and GGT activity

Franzini et al. identified four GGT fractions in serum: big-GGT, medium-GGT, small-GGT and free-GGT fractions [24] and recently showed that the big-GGT fraction corresponds to exosomal GGT [25]. Here we subjected serum of a healthy individual to size exclusion chromatography (SEC) and measured GGT activity and CD9 expression in each fraction. The level of CD9 was determined by a sandwich ELISA. SEC yielded a minor peak and a major peak of GGT activity (Fig. 4a). The minor peak spanning fractions 4 to 9 was positive for CD9, indicating that GGT activity was detected in serum exosomes. Western blot analysis of the fractions 3 to 10 obtained from the same healthy individual revealed the co-presence of GGT1 large and small subunits with CD9 (Fig. 4b). We also subjected exosomes isolated from human serum by differential centrifugation to OptiPrep density gradient centrifugation. The results showed that GGT1 large and small subunits were detected only in fraction 9 that was positive for CD9 (Fig. 5a), indicating that serum exosomes isolated by differential centrifugation is free of contamination with other GGT forms such as medium-GGT, small-GGT and free-GGT. Lastly, we isolated serum exosomes by differential centrifugation from BPH (*n* = 4) and PC (*n* = 8) patients and determined GGT1 expression (Fig. 5b) as well as GGT activity. Spearman’s rank correlation analysis revealed correlation of GGT activity with the signal intensity of GGT1 large subunit in serum exosomes (Fig. 5c). These results provided the basis for measuring GGT activity in serum exosomes isolated by differential centrifugation using the gGlu-HMRG probe.

### No association between serum exosomal GGT activity and CRPC

Since we identified GGT1 as an exosomal marker upregulated in castration-resistant C4–2 and bone metastatic C4–2B cells, we hypothesized that GGT activity in serum exosomes could be a marker for CRPC and/or bone metastasis. We isolated exosomes by differential centrifugation from serum of patients with PC and measured GGT activity using the gGlu-HMRG probe. Contrary to our expectation, however, serum exosomal GGT activity exhibited no difference between PC patients with (*n* = 6, PSA: 7.46–585.70 ng/mL) and without (*n* = 35, PSA: 4.20–549.39 ng/mL) castration-resistance (Additional file 2: Fig. S1). The association of serum exosomal GGT activity with bone metastasis was not examined due to limited number of appropriate patients. These results suggested that GGT activity in serum exosomes isolated by differential centrifugation would have little or no potential as a marker for CRPC in PC patients.

### Increased serum exosomal GGT activity in PC patients than in BPH patients

**Fig. 6:**
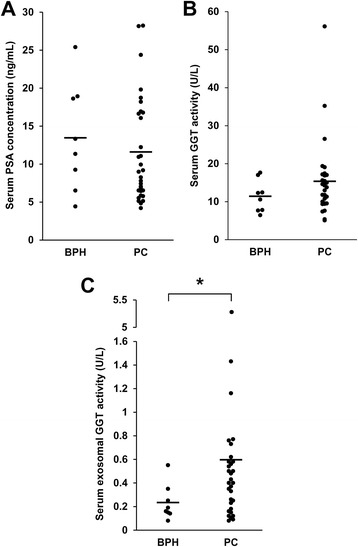
GGT activity in exosomes isolated by differential centrifugation from serum of PC and BPH patients. Exosomes were isolated by differential centrifugation from serum (210 μL) of PC (*n* = 31) and BPH (*n* = 8) patients. GGT activity was determined by incubation with gGlu-HMRG at room temperature for 1 h and measurement of fluorescence intensity (Ex/Em 490/520 nm) using microplate reader. Patient groups were compared for serum PSA concentration (**a**), serum GGT activity (**b**) and serum exosomal GGT activity (**c**). *p < 0.05, compared with BPH patients

It has been reported that serum GGT activity was increased in certain types of cancer [22] and thus we measured serum GGT activity as well as serum exosomal GGT activity in patients with BPH (*n* = 8, PSA: 4.42–25.40 ng/mL) and PC patients (*n* = 31, PSA: 4.20–28.23 ng/mL). The results showed that there was no statistical difference in the serum PSA concentration (Fig. 6a) and serum GGT activity (Fig. 6b) between two patient groups. In contrast, GGT activity in serum exosomes was significantly increased in patients with PC compared to those with BPH (Fig. 6c). These results suggested that serum exosomal GGT activity but not serum GGT activity could be a biomarker to distinguish PC patients from BPH patients, both of which exhibited similar serum PSA levels.

### Increased GGT1 expression in PC tissues than in BPH tissues

**Fig. 7:**
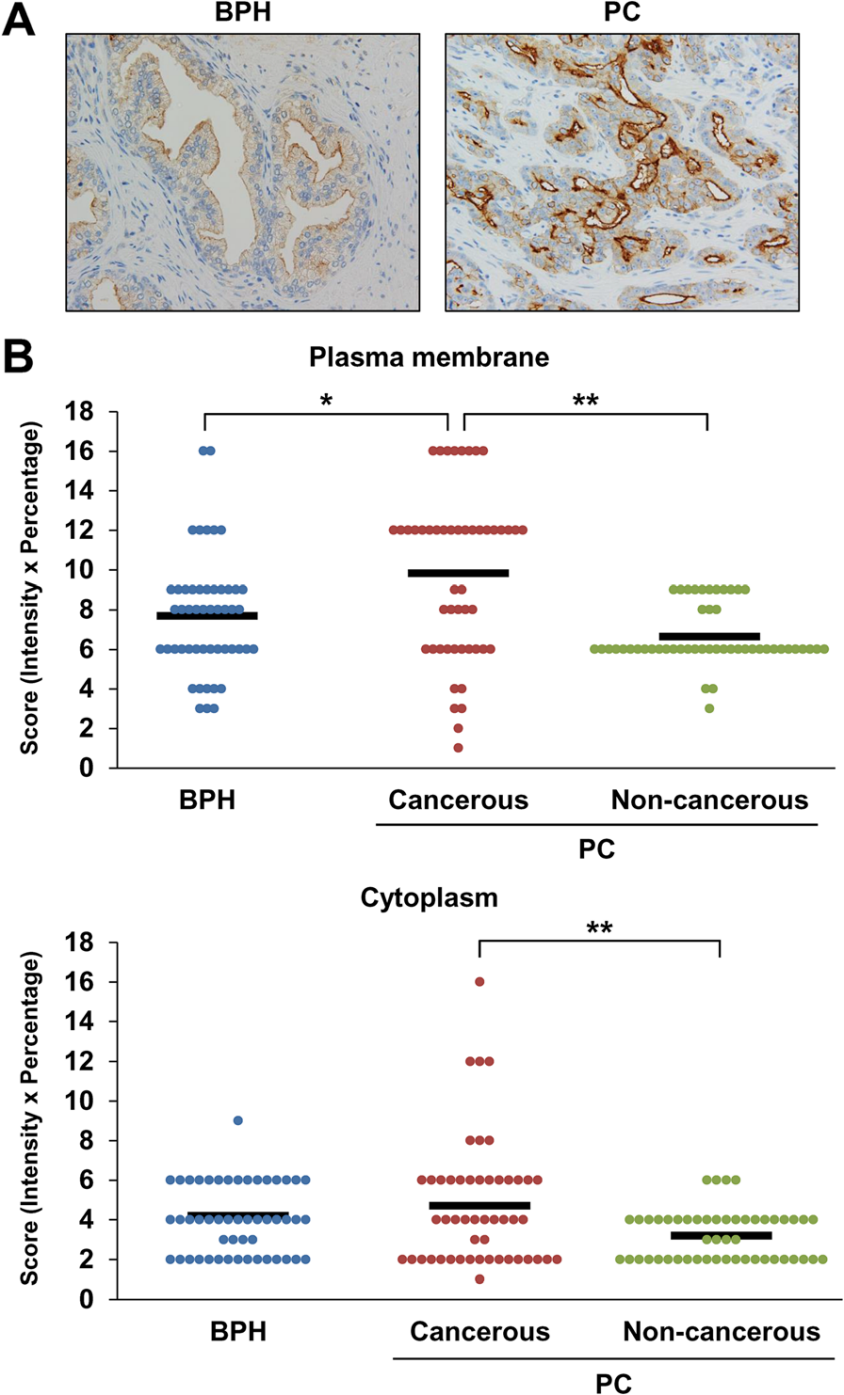
Immunohistochemical analysis of GGT1 in PC and BPH tissues. Formalin-fixed paraffin-embedded biopsies and surgically resected tissue specimens from PC (*n* = 50) and BPH (*n* = 50) patients were stained for GGT1. **a** Representative images in BPH and PC tissues are shown. Original magnification ×400. **b** GGT1 expression on the plasma membrane and in the cytoplasm of cancerous and non-cancerous lesions of PC and BPH tissues is expressed as a score calculated by multiplying the intensity score with the percentage score. *p < 0.01, compared with BPH tissues. **p < 0.001, compared with the non-cancerous lesion

Elevated serum exosomal GGT activity in PC patients compared with BPH patients suggested the possibility that GGT1 expression might be increased in PC tissues than in BPH tissues. In order to prove our hypothesis, we performed immunohistochemical staining of GGT1 using formalin-fixed paraffin-embedded biopsies and surgically resected tissue specimens from PC and BPH patients. The clinical and pathological profile of patients is shown in Additional file 3: Table S2. In BPH tissues, prostatic glands showed weak apical expression for GGT1 (Fig. 7a). In PC tissues, cancer cells showed cytoplasmic and membranous expression for GGT1 and background noncancerous prostatic glands showed weak apical expression. In order to evaluate GGT1 expression on the plasma membrane and in the cytoplasm, staining was scored for the intensity and percentage and then both scores were multiplied (Fig. 7b). GGT1 expression on the plasma membrane was increased in PC tissues compared with BPH tissues (*p* < 0.01), whereas that in the cytoplasm showed no statistically significant difference. When GGT1 expression was compared within the PC tissues, membranous and cytoplasmic expression was higher in the cancerous lesion than in the non-cancerous lesion (*p* < 0.001 and *p* < 0.001, respectively). There were no statistical differences between GGT1 expression and Gleason score. These results indicated that GGT1 expression was elevated in PC tissues than in BPH tissues, supporting our findings of increased serum exosomal GGT activity in PC patients.

## Discussion

Based on proteomic analysis of exosomes isolated from PC cell lines by differential centrifugation, we identified GGT1 as a potential exosomal marker for PC. GGT also known as gamma-glutamyl transpeptidase is an enzyme that transfers a gamma-glutamyl group from GSH and other γ-glutamyl compounds to amino acids or dipeptides. GSH is abundant in the cells and plays important roles in protection from oxidative stress and maintenance of the redox status [26]. GGT initiates the degradation of extracellular GSH, resulting in production of cysteinylglycine and glutamate. Cysteinylglycine is then hydrolyzed by cell surface dipeptidase to generate glycine and cysteine. The degraded amino acids are used for de novo synthesis of GSH. In normal human tissues, strong GGT immunoreactivity was observed on the surface of renal proximal tubule cells, hepatic bile canaliculi and capillary endothelial cells within the nervous system [27]. Secretory or absorptive cells in sweat glands, prostate, salivary gland ducts, bile ducts, pancreatic acini, intestinal crypts and testicular tubules were also GGT-positive. Among a family of GGT genes in the human genome [28], GGT1, which is generally referred to as GGT, is shown to be involved in GSH metabolism [29].

Elevation of GGT expression has been reported for a number of cancers including colon, ovary and liver cancer, astrocytic glioma, soft tissue sarcoma, melanoma and leukemia [22]. A comprehensive analysis of GGT expression showed that most tumors derived from tissues expressing GGT were positive for GGT and that lung and ovary cancer derived from GGT-negative epithelia also expressed GGT [22]. GGT expression was linked to unfavorable prognostic signs in breast cancer, but no correlation between GGT expression and standard clinical pathological parameters has been found in prostatic, colorectal and breast cancer [22].

Upregulation of GGT expression in cancer has been considered to protect cancer cells against oxidative stress by increasing the intracellular GSH level and thereby support their growth and survival [30]. However, it was also demonstrated that the metabolism of GSH by GGT can exert pro-oxidant effects [31]. Upregulation of GGT may impose an increased oxidative burden on the cell, resulting in GSH consumption and a decrease of cellular GSH stores. The persistent production of ROS caused by increased GGT expression may contribute to genetic instability and tumor progression [32].

Serum GGT activity is commonly used as a marker for liver, gallbladder and biliary tract diseases especially alcoholic liver disease because it is particularly sensitive to alcohol consumption [33]. On the other hand, a positive association of serum GGT activity with the risk of cancer [34, 35] as well as cardiovascular diseases and metabolic syndrome [36] has been reported. Furthermore, serum GGT levels were found to be higher in hepatocellular carcinoma patients with poorly differentiated tumors as compared to those with well and moderately differentiated tumors [37]. In renal cell carcinoma, serum GGT activity was reported to be increased in most of patients with metastasis, while it was normal in majority of patients with localized tumor [38].

Franzini et al. performed gel filtration chromatography followed by postcolumn reaction with a fluorescent GGT substrate, gamma-glutamyl-7-amido-4-methylcoumarin (γGluAMC) and identified four GGT fractions in serum: big-GGT, medium-GGT, small-GGT and free-GGT fractions of different molecular weight (molecular masses >2000 kDa, 940 kDa, 140 kDa and 70 kDa, respectively) [24]. The authors demonstrated that b-GGT increased in non-alcoholic fatty liver disease (NAFLD) but not in chronic hepatitis C (CHC) and that b-GGT/s-GGT ratio showed the highest diagnostic accuracy for distinguishing NAFLD and CHC [39]. They also showed that the big-GGT fraction corresponds to serum exosomal GGT [25].

In order to determine GGT activity on exosomes, we used a newly reported fluorescence probe, gGlu-HMRG, which is activated by rapid one-step cleavage of glutamate with GGT [20]. This probe was developed to detect cancers cells during surgical and endoscopic procedures, taking advantage of its activation by GGT that is present on the cell surface. In vivo imaging of superficial head and neck squamous cell carcinoma and beast, lung and colorectal cancer using gGlu-HMRG has been reported [40–43]. In vitro activation of gGlu-HMRG was also shown in human ovarian cancer cell lines [20].

In the present study, we first showed correlation of GGT1 expression with GGT activity in cell lysates and exosomes. Second, we separated human serum by SEC and demonstrated that the minor peak that was positive for CD9 contained GGT1 large and small subunits as well as GGT activity and that the major peak was presumably comprised of medium-GGT, small-GGT and free-GGT fractions other than big-GGT or exosomal GGT fraction. Third, we subjected exosomes isolated from human serum by differential centrifugation to OptiPrep density gradient centrifugation and confirmed that exosomes isolated from human serum by differential centrifugation is free of contamination with other GGT forms. Lastly, based on these findings, we measured serum exosomal GGT activity in patients. Despite the fact that GGT1 was upregulated in exosomes isolated from androgen-independent C4–2 and bone metastatic C4–2B cells, there was no difference between PC patients with and without castration-resistance. Unexpectedly, we found that serum exosomal GGT activity was significantly higher in PC patients than in BPH patients.

In support of our findings of increased serum exosomal GGT activity in PC patients, GGT1 expression was elevated in PC tissues compared with BPH tissues. A previous report showed that the majority of neoplastic cells were positive for GGT1 in most of PC [44]. In the present study, we demonstrated that there was a significant difference in GGT1 expression between PC and BPH tissues. Furthermore, cancer cells showed stronger expression for GGT1 in the cytoplasm and membrane than background noncancerous prostatic glands. These results suggested that prostatic cancer cells may produce more exosomes expressing GGT1. The underlying mechanism that is responsible for overexpression of GGT1 in PC remains to be elucidated.

Numerous reports have proposed potential markers for PC based on pathological and clinical research [45]. More recently identified PC markers include prostate cancer antigen 3 (PCA3) [46], TMPRSS2-ERG fusion gene [47] and their combined use [48]. Although there have been a limited number of reports describing exosomal miRNA as a marker for PC [49], we and others have reported exosomal protein markers that would be helpful to diagnose PC (PSMA), taxane-resistant CRPC (P-gp) and progression and aggressiveness of PC (integrin β4 and vinculin) [12–16]. This is the first report that described serum exosomal GGT1 expression or GGT activity as a potential marker to diagnose PC.

PSA is a commonly used marker for PC, but it cannot distinguish PC from BPH when the levels are similar [9, 10]. In the present study, we measured serum exosomal GGT activity as well as serum GGT activity and serum PSA level in two patient groups. As shown in Additional file 4: Fig. S2, the AUC of serum exosomal GGT activity was 0.714 (95% CI between 0.535 and 0.892), while that of serum GGT activity was 0.621 (95% CI between 0.396 and 0.846) and that of serum PSA concentration was 0.601 (95% CI between 0.361 and 0.841). These results suggest that serum exosomal GGT activity but not serum GGT activity could be a biomarker to differentiate PC patients from BPH patients, both of which exhibit similar serum PSA levels.

Although we have demonstrated the potential of serum exosomal GGT activity for differential diagnosis of PC and BPH, the current detection system has limitations for clinical application, because differential centrifugation is required to measure the activity. It is also worth noting that GGT1 is expressed in normal tissues and thus serum exosomes isolated by differential centrifugation may contain those derived from various tissues. We and others have recently demonstrated that exosomes derived from PC could be isolated by immunocapture with anti-PSMA antibody [12, 13]. The development of an antibody with a higher affinity for PSMA and its use would enable us to increase the specificity and sensitivity of serum exosomal GGT activity as a marker for PC.

The usefulness of serum exosomal GGT activity as a maker to diagnose PC needs to be validated in large-scale clinical studies. Since serum GGT activity has been implicated in a variety of diseases by clinical and epidemiological studies [34–36, 50], it would be of great interest to test if serum exosomal GGT activity is superior to serum GGT activity in other diseases than PC. Nevertheless, in order to conduct large-scale studies, a simple and rapid detection system remains to be established, which would make it possible to evaluate the potential of serum exosomal GGT activity as prognostic as well as diagnostic markers in prospective clinical studies. Finally, it is also of great importance to understand the properties and roles of GGT1 on exosomes in serum of patients.

## Conclusions

We demonstrated that GGT activity in serum exosomes was significantly higher in PC patients than in BPH patients, which was supported by increased GGT1 expression in PC tissues compared with BPH tissues. Serum exosomal GGT activity could be a useful marker to diagnose PC or to distinguish PC from BPH and possibly to diagnose other types of cancer with increased GGT1 expression.

## Additional files

Additional file 1: Table S1. List of differentially expressed proteins. (PDF 97 kb)

Additional file 2: Fig. S1. GGT activity in exosomes isolated by differential centrifugation from serum of PC patients. (PDF 40 kb)

Additional file 3: Table S2. Patient characteristics. (PDF 77 kb)

Additional file 4: Fig. S2. ROC curve analysis of PC and BPH patients. (PDF 30 kb)

## Abbreviations

BPH: Benign prostatic hyperplasia
CHC: Chronic hepatitis C
CRPC: Castration-resistant prostate cancer
EV: Extracellular vesicles
gGlu-HMRG: γ-glutamyl hydroxymethyl rhodamine green
GGT1: Gamma-glutamyltransferase 1
GSH: Glutathione
MDR1: Multi-drug resistance protein 1
NAFLD: Non-alcoholic fatty liver disease
PC: Prostate cancer
PCA3: Prostate cancer antigen 3
P-gp: P-glycoprotein
PSA: Prostate-specific antigen
PSMA: Prostate-specific membrane antigen
PVDF: Polyvinylidene difluoride
γGluAMC: gamma-glutamyl-7-amido-4-methylcoumarin
