# Supplementary material for: Gamma-glutamyltransferase activity in exosomes as a potential marker for prostate cancer
Source: BMC Cancer. 2017 May 5;17:316. doi: 10.1186/s12885-017-3301-x (PMC5420129; doi:10.1186/s12885-017-3301-x)
Supplement: Supplementary file 1 — List of differentially expressed proteins. (PDF 97 kb) [file 12885_2017_3301_MOESM1_ESM.pdf]

**Table S1. List of differentially expressed proteins**

| Accession Number | Protein Name | Gene Symbol | C4 /LNCaP | C4-2 /LNCaP | C4-2B /LNCaP |
|---|---|---|---|---|---|
| P05106 | Integrin beta-3 | ITGB3 | 0.78 | 1.50 | 2.00 |
| O60716 | Catenin delta-1 | CTNND1 | 1.10 | 1.58 | 1.94 |
| P35221 | Catenin alpha-1 | CTNNA1 | 0.97 | 1.51 | 1.77 |
| P02765 | Alpha-2-HS-glycoprotein | AHSG | 0.96 | 1.19 | 1.71 |
| P19440 | Gamma-glutamyltranspeptidase 1 | GGT1 | 0.82 | 1.56 | 1.63 |
| P61224 | Ras-related protein Rap-1b | RAP1B | 0.75 | 1.25 | 1.58 |
| P62258 | 14-3-3 protein epsilon | YWHAE | 1.66 | 1.29 | 1.54 |
| P53985 | Monocarboxylate transporter 1 | SLC16A1 | 1.13 | 1.56 | 1.51 |
| P04406 | Glyceraldehyde-3-phosphate dehydrogenase | GAPDH | 1.51 | 1.28 | 1.41 |
| Q86X29 | Lipolysis-stimulated lipoprotein receptor | LSR | 0.58 | 1.24 | 1.41 |
| Q8TDB8 | Solute carrier family 2, facilitated glucose transporter member 14 | SLC2A14 | 0.93 | 1.08 | 1.41 |
| P08514 | Integrin alpha-IIb | ITGA2B | 0.85 | 1.31 | 1.39 |
| P00742 | Coagulation factor X | F10 | 0.90 | 1.11 | 1.33 |
| P21333 | Filamin-A | FLNA | 0.78 | 1.13 | 1.29 |
| P07195 | L-lactate dehydrogenase B chain | LDHB | 1.46 | 1.21 | 1.27 |
| P12277 | Creatine kinase B-type | CKB | 0.89 | 1.16 | 1.26 |
| O00159 | Myosin-Ic | MYO1C | 0.90 | 1.33 | 1.26 |
| P01024 | Complement C3 | C3 | 1.11 | 1.34 | 1.26 |
| P13224 | Platelet glycoprotein Ib beta chain | GP1BB | 0.89 | 1.17 | 1.24 |
| P08758 | Annexin A5 | ANXA5 | 0.74 | 0.99 | 1.22 |
| P35222 | Catenin beta-1 | CTNNB1 | 0.82 | 1.27 | 1.21 |
| Q04917 | 14-3-3 protein eta | YWHAH | 1.05 | 1.24 | 1.21 |
| P04899 | Guanine nucleotide-binding protein G(i) subunit alpha-2 | GNAI2 | 1.09 | 1.11 | 1.20 |
| P00533 | Epidermal growth factor receptor | EGFR | 0.95 | 1.16 | 1.19 |
| Q5ZPR3 | CD276 antigen | CD276 | 0.95 | 1.01 | 1.19 |
| P49327 | Fatty acid synthase | FASN | 1.43 | 1.20 | 1.18 |
| P54709 | Sodium/potassium-transporting ATPase subunit beta-3 | ATP1B3 | 1.42 | 0.97 | 1.18 |
| P01023 | Alpha-2-macroglobulin | A2M | 0.80 | 1.41 | 1.18 |
| P31946 | 14-3-3 protein beta/alpha | YWHAB | 1.14 | 1.27 | 1.18 |
| Q92928 | Putative Ras-related protein Rab-1C | RAB1C | 1.02 | 1.03 | 1.17 |
| P52565 | Rho GDP-dissociation inhibitor 1 | ARHGDIA | 1.18 | 1.18 | 1.17 |
| Q5JWF2 | Guanine nucleotide-binding protein G(s) subunit alpha isoforms XLas | GNAS | 0.96 | 1.12 | 1.15 |
| P06744 | Glucose-6-phosphate isomerase | GPI | 1.07 | 1.07 | 1.13 |
| P20742 | Pregnancy zone protein | PZP | 0.96 | 1.42 | 1.12 |
| Q9NZN3 | EH domain-containing protein 3 | EHD3 | 0.92 | 1.05 | 1.11 |
| P06396 | Gelsolin | GSN | 0.84 | 1.09 | 1.11 |
| P61981 | 14-3-3 protein gamma | YWHAG | 1.15 | 1.10 | 1.10 |
| P04792 | Heat shock protein beta-1 | HSPB1 | 1.08 | 1.11 | 1.10 |
| P84077 | ADP-ribosylation factor 1 | ARF1 | 1.07 | 0.94 | 1.10 |
| P50995 | Annexin A11 | ANXA11 | 1.03 | 0.95 | 1.10 |
| P01111 | GTPase NRas | NRAS | 1.05 | 1.00 | 1.10 |
| P26038 | Moesin | MSN | 0.98 | 1.03 | 1.09 |
| P62937 | Peptidyl-prolyl cis-trans isomerase A | PPIA | 1.41 | 0.98 | 1.09 |
| P02787 | Serotransferrin | TF | 1.00 | 0.99 | 1.09 |
| P35613-2 | Isoform 2 of Basigin | BSG | 0.98 | 1.04 | 1.07 |
| P68871 | Hemoglobin subunit beta | HBB | 0.99 | 0.96 | 1.07 |
| P14174 | Macrophage migration inhibitory factor | MIF | 1.20 | 1.03 | 1.07 |
| P63104 | 14-3-3 protein zeta/delta | YWHAZ | 0.99 | 1.07 | 1.06 |
| Q15907 | Ras-related protein Rab-11B | RAB11B | 0.99 | 1.05 | 1.06 |
| Q00796 | Sorbitol dehydrogenase | SORD | 0.96 | 0.99 | 1.06 |
| Q9H5V8 | CUB domain-containing protein 1 | CDCP1 | 1.16 | 1.05 | 1.06 |
| Q01650 | Large neutral amino acids transporter small subunit 1 | SLC7A5 | 0.63 | 1.12 | 1.06 |
| P62070 | Ras-related protein R-Ras2 | RRAS2 | 0.93 | 1.09 | 1.06 |
| P60953-2 | Isoform Placental of Cell division control protein 42 homolog | CDC42 | 1.03 | 1.06 | 1.06 |
| P04075 | Fructose-bisphosphate aldolase A | ALDOA | 0.91 | 1.10 | 1.05 |
| P69905 | Hemoglobin subunit alpha | HBA1 | 0.96 | 0.98 | 1.05 |
| P02749 | Beta-2-glycoprotein 1 | APOH | 0.86 | 0.91 | 1.05 |
| P11166 | Solute carrier family 2, facilitated glucose transporter member 1 | SLC2A1 | 0.98 | 0.97 | 1.04 |
| P61586 | Transforming protein RhoA | RHOA | 1.04 | 1.01 | 1.04 |
| P12259 | Coagulation factor V | F5 | 0.98 | 1.00 | 1.04 |
| P14923 | Junction plakoglobin | JUP | 1.00 | 0.97 | 1.03 |
| P11233 | Ras-related protein Ral-A | RALA | 0.96 | 1.01 | 1.03 |
| P07737 | Profilin-1 | PFN1 | 1.07 | 0.98 | 1.03 |
| P49006 | MARCKS-related protein | MARCKSL1 | 1.05 | 1.00 | 1.03 |
| P35241 | Radixin | RDX | 1.01 | 1.00 | 1.03 |

| | | | | | |
|---|---|---|---|---|---|
| P60174 | Triosephosphate isomerase | TPI1 | 1.04 | 1.02 | 1.02 |
| P05556 | Integrin beta-1 | ITGB1 | 0.97 | 0.96 | 1.02 |
| O14672 | Disintegrin and metalloproteinase domain-containing protein 10 | ADAM10 | 0.97 | 1.03 | 1.02 |
| P0C0L5 | Complement C4-B | C4B | 1.08 | 1.06 | 1.02 |
| P60981 | Destrin | DSTN | 1.10 | 0.90 | 1.02 |
| Q14126 | Desmoglein-2 | DSG2 | 1.01 | 0.96 | 1.01 |
| P16422 | Epithelial cell adhesion molecule | EPCAM | 1.03 | 0.98 | 1.01 |
| P63000 | Ras-related C3 botulinum toxin substrate 1 | RAC1 | 0.98 | 1.03 | 1.01 |
| Q9Y490 | Talin-1 | TLN1 | 0.90 | 0.96 | 1.01 |
| P00734 | Prothrombin | F2 | 0.90 | 0.96 | 1.01 |
| P14618 | Pyruvate kinase isozymes M1/M2 | PKM2 | 1.06 | 1.00 | 1.00 |
| P62879 | Guanine nucleotide-binding protein G(I)/G(S)/G(T) subunit beta-2 | GNB2 | 0.93 | 0.97 | 1.00 |
| P20073 | Annexin A7 | ANXA7 | 1.13 | 0.97 | 1.00 |
| P04083 | Annexin A1 | ANXA1 | 0.85 | 1.07 | 1.00 |
| Q08722 | Leukocyte surface antigen CD47 | CD47 | 0.96 | 1.01 | 1.00 |
| P06733 | Alpha-enolase | ENO1 | 1.08 | 0.99 | 0.99 |
| P02786 | Transferrin receptor protein 1 | TFRC | 1.10 | 1.06 | 0.99 |
| Q8IWA5-2 | Isoform B of Choline transporter-like protein 2 | SLC44A2 | 0.90 | 1.04 | 0.99 |
| Q9Y446 | Plakophilin-3 | PKP3 | 0.91 | 1.01 | 0.99 |
| P51149 | Ras-related protein Rab-7a | RAB7A | 1.04 | 1.06 | 0.99 |
| P00558 | Phosphoglycerate kinase 1 | PGK1 | 1.04 | 0.99 | 0.99 |
| P23634 | Plasma membrane calcium-transporting ATPase 4 | ATP2B4 | 0.97 | 0.96 | 0.99 |
| P05023 | Sodium/potassium-transporting ATPase subunit alpha-1 | ATP1A1 | 1.54 | 0.96 | 0.98 |
| P07900 | Heat shock protein HSP 90-alpha | HSP90AA1 | 1.07 | 0.95 | 0.98 |
| P18206 | Vinculin | VCL | 0.97 | 0.90 | 0.98 |
| P23528 | Cofilin-1 | CFL1 | 1.06 | 0.89 | 0.98 |
| P15311 | Ezrin | EZR | 1.05 | 0.97 | 0.98 |
| P50395 | Rab GDP dissociation inhibitor beta | GDI2 | 1.07 | 0.98 | 0.98 |
| P32119 | Peroxiredoxin-2 | PRDX2 | 1.02 | 0.97 | 0.98 |
| P13639 | Elongation factor 2 | EEF2 | 1.04 | 0.96 | 0.97 |
| P62330 | ADP-ribosylation factor 6 | ARF6 | 0.99 | 0.83 | 0.97 |
| Q9UNF0 | Protein kinase C and casein kinase substrate in neurons protein 2 | PACSIN2 | 1.01 | 0.91 | 0.97 |
| Q9P2B2 | Prostaglandin F2 receptor negative regulator | PTGFRN | 0.93 | 0.96 | 0.96 |
| P08238 | Heat shock protein HSP 90-beta | HSP90AB1 | 0.90 | 0.92 | 0.96 |
| O15394 | Neural cell adhesion molecule 2 | NCAM2 | 0.98 | 0.96 | 0.96 |
| Q969P0 | Immunoglobulin superfamily member 8 | IGSF8 | 1.04 | 0.91 | 0.96 |
| P12830 | Cadherin-1 | CDH1 | 1.09 | 1.08 | 0.96 |
| P02751 | Fibronectin | FN1 | 0.10 | 2.11 | 0.96 |
| P09543 | 2',3'-cyclic-nucleotide 3'-phosphodiesterase | CNP | 0.96 | 0.93 | 0.96 |
| Q8NFT2 | Metalloreductase STEAP2 | STEAP2 | 1.04 | 0.96 | 0.95 |
| P31939 | Bifunctional purine biosynthesis protein PURH | ATIC | 0.94 | 0.88 | 0.95 |
| P05026 | Sodium/potassium-transporting ATPase subunit beta-1 | ATP1B1 | 0.90 | 0.91 | 0.94 |
| P62873 | Guanine nucleotide-binding protein G(I)/G(S)/G(T) subunit beta-1 | GNB1 | 0.91 | 0.99 | 0.94 |
| P50502 | Hsc70-interacting protein | ST13 | 1.06 | 0.87 | 0.94 |
| Q12846 | Syntaxin-4 | STX4 | 0.96 | 1.05 | 0.94 |
| P08473 | Neprilysin | MME | 0.27 | 0.90 | 0.93 |
| P22392-2 | Isoform NM23-LV of Nucleoside diphosphate kinase B | NME2 | 1.19 | 1.04 | 0.93 |
| P11940 | Polyadenylate-binding protein 1 | PABPC1 | 0.98 | 0.88 | 0.93 |
| P08133 | Annexin A6 | ANXA6 | 0.96 | 0.91 | 0.92 |
| P02748 | Complement component C9 | C9 | 0.82 | 0.99 | 0.91 |
| P68104 | Elongation factor 1-alpha 1 | EEF1A1 | 1.46 | 0.78 | 0.90 |
| P55072 | Transitional endoplasmic reticulum ATPase | VCP | 0.92 | 0.85 | 0.90 |
| P60842 | Eukaryotic initiation factor 4A-I | EIF4A1 | 1.00 | 0.92 | 0.90 |
| P60660 | Myosin light polypeptide 6 | MYL6 | 1.10 | 0.94 | 0.90 |
| P62158 | Calmodulin | CALM1 | 0.98 | 0.93 | 0.90 |
| P10599 | Thioredoxin | TXN | 1.14 | 0.86 | 0.90 |
| P08195 | 4F2 cell-surface antigen heavy chain | SLC3A2 | 0.63 | 0.96 | 0.88 |
| O43175 | D-3-phosphoglycerate dehydrogenase | PHGDH | 1.04 | 0.94 | 0.88 |
| P27348 | 14-3-3 protein theta | YWHAQ | 1.12 | 0.95 | 0.87 |
| P63261 | Actin, cytoplasmic 2 | ACTG1 | 0.77 | 0.93 | 0.86 |
| P61026 | Ras-related protein Rab-10 | RAB10 | 0.88 | 0.94 | 0.86 |
| P68371 | Tubulin beta-2C chain | TUBB2C | 1.11 | 0.83 | 0.83 |
| O75340 | Programmed cell death protein 6 | PDCD6 | 1.09 | 0.79 | 0.82 |
| P51153 | Ras-related protein Rab-13 | RAB13 | 0.79 | 0.82 | 0.82 |
| Q9UHE8 | Metalloreductase STEAP1 | STEAP1 | 0.90 | 0.79 | 0.81 |
| Q06830 | Peroxiredoxin-1 | PRDX1 | 1.12 | 0.55 | 0.78 |
| Q99880 | Histone H2B type 1-L | HIST1H2BL | 1.10 | 1.07 | 0.78 |
| Q15758 | Neutral amino acid transporter B(0) | SLC1A5 | 0.97 | 0.79 | 0.77 |

| | | | | | |
|---|---|---|---|---|---|
| Q04609 | Glutamate carboxypeptidase 2 | FOLH1 | 1.21 | 0.71 | 0.73 |
| O00560 | Syntenin-1 | SDCBP | 0.80 | 0.72 | 0.72 |
| O00560-2 | Isoform 2 of Syntenin-1 | SDCBP | 0.74 | 0.77 | 0.72 |
| Q00610 | Clathrin heavy chain 1 | CLTC | 0.69 | 1.36 | 0.70 |
| P68363 | Tubulin alpha-1B chain | TUBA1B | 0.95 | 0.61 | 0.65 |
| P21926 | CD9 antigen | CD9 | 0.43 | 0.71 | 0.64 |
| Q9BTM1 | Histone H2A.J | H2AFJ | 1.61 | 2.11 | 0.63 |
| P04004 | Vitronectin | VTN | 1.63 | 1.34 | 0.62 |
| Q8WUM4 | Programmed cell death 6-interacting protein | PDCD6IP | 0.81 | 0.52 | 0.61 |
| P08107 | Heat shock 70 kDa protein 1A/1B | HSPA1A | 0.60 | 0.63 | 0.60 |
| P29966 | Myristoylated alanine-rich C-kinase substrate | MARCKS | 1.42 | 0.57 | 0.56 |
| Q08431 | Lactadherin | MFGE8 | 1.17 | 0.42 | 0.54 |
| P11142 | Heat shock cognate 71 kDa protein | HSPA8 | 0.85 | 0.40 | 0.49 |
| Q9BUD6 | Spondin-2 | SPON2 | 3.08 | 0.91 | 0.48 |
| P62988 | Ubiquitin | RPS27A | 0.74 | 0.44 | 0.47 |
| O75131 | Copine-3 | CPNE3 | 0.99 | 0.56 | 0.46 |
| P62805 | Histone H4 | HIST1H4A | 1.49 | 2.42 | 0.42 |
| P07437 | Tubulin beta chain | TUBB | 0.77 | 0.30 | 0.29 |
| P13645 | Keratin, type I cytoskeletal 10 | KRT10 | 0.05 | 0.39 | 0.16 |
| P04264 | Keratin, type II cytoskeletal 1 | KRT1 | 0.06 | 0.22 | 0.10 |
