# Supplementary material for: Gamma-glutamyltransferase activity in exosomes as a potential marker for prostate cancer
Source: BMC Cancer. 2017 May 5;17:316. doi: 10.1186/s12885-017-3301-x (PMC5420129; doi:10.1186/s12885-017-3301-x)
Supplement: Supplementary file 2 — GGT activity in exosomes isolated by differential centrifugation from serum of PC patients. (PDF 40 kb) [file 12885_2017_3301_MOESM2_ESM.pdf]

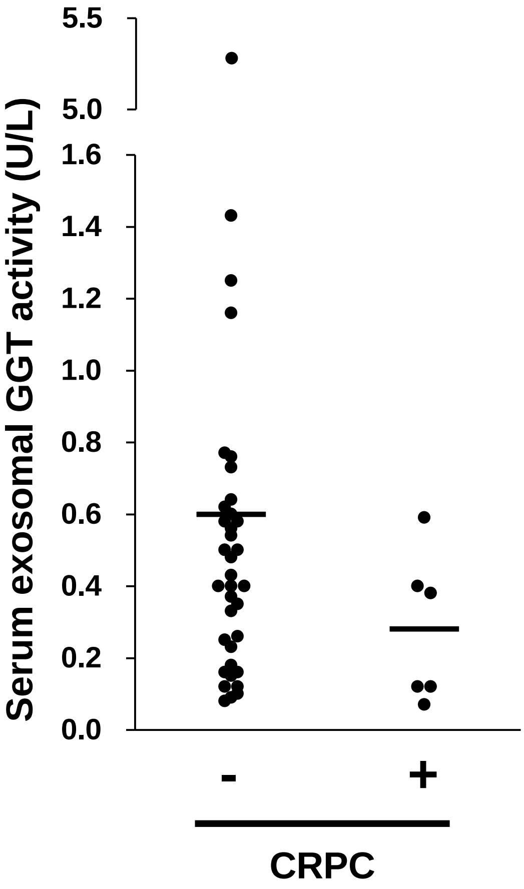

Figure S1. GGT activity in exosomes isolated by differential centrifugation from serum of PC patients. Exosomes were isolated by differential centrifugation from serum (210 μL) of PC patients without (n=35) and with (n=6) castration-resistance. GGT activity in exosomes was determined by incubation with gGlu-HMRG at room temperature for 1 h and measurement of fluorescence intensity (Ex/Em 490/520 nm) using microplate reader.
