# Supplementary material for: Gamma-glutamyltransferase activity in exosomes as a potential marker for prostate cancer
Source: BMC Cancer. 2017 May 5;17:316. doi: 10.1186/s12885-017-3301-x (PMC5420129; doi:10.1186/s12885-017-3301-x)
Supplement: Supplementary file 3 — Patient characteristics. (PDF 77 kb) [file 12885_2017_3301_MOESM3_ESM.pdf]

Table S2. Patient characteristics

	BPH		PC	
Number	50		50	
Median age (IQR)	73.5	(69-77)	75	(70.5-79)
T stage (%)				
T1	-		1	(2)
T2	-		39	(78)
T3	-		10	(20)
N stage (%)				
N0	-		50	(100)
M stage (%)				
M0	-		50	(100)
Median PSA (ng/mL) (IQR)	8.06	(5.70-12.70)	9.87	(6.67-14.96)
Gleason sum (%)				
6	-		6	(12)
7	-		26	(52)
8-10	-		18	(36)

IQR, Interquartile range
