# Supplementary material for: Gamma-glutamyltransferase activity in exosomes as a potential marker for prostate cancer
Source: BMC Cancer. 2017 May 5;17:316. doi: 10.1186/s12885-017-3301-x (PMC5420129; doi:10.1186/s12885-017-3301-x)
Supplement: Supplementary file 4 — ROC curve analysis of PC and BPH patients. (PDF 30 kb) [file 12885_2017_3301_MOESM4_ESM.pdf]

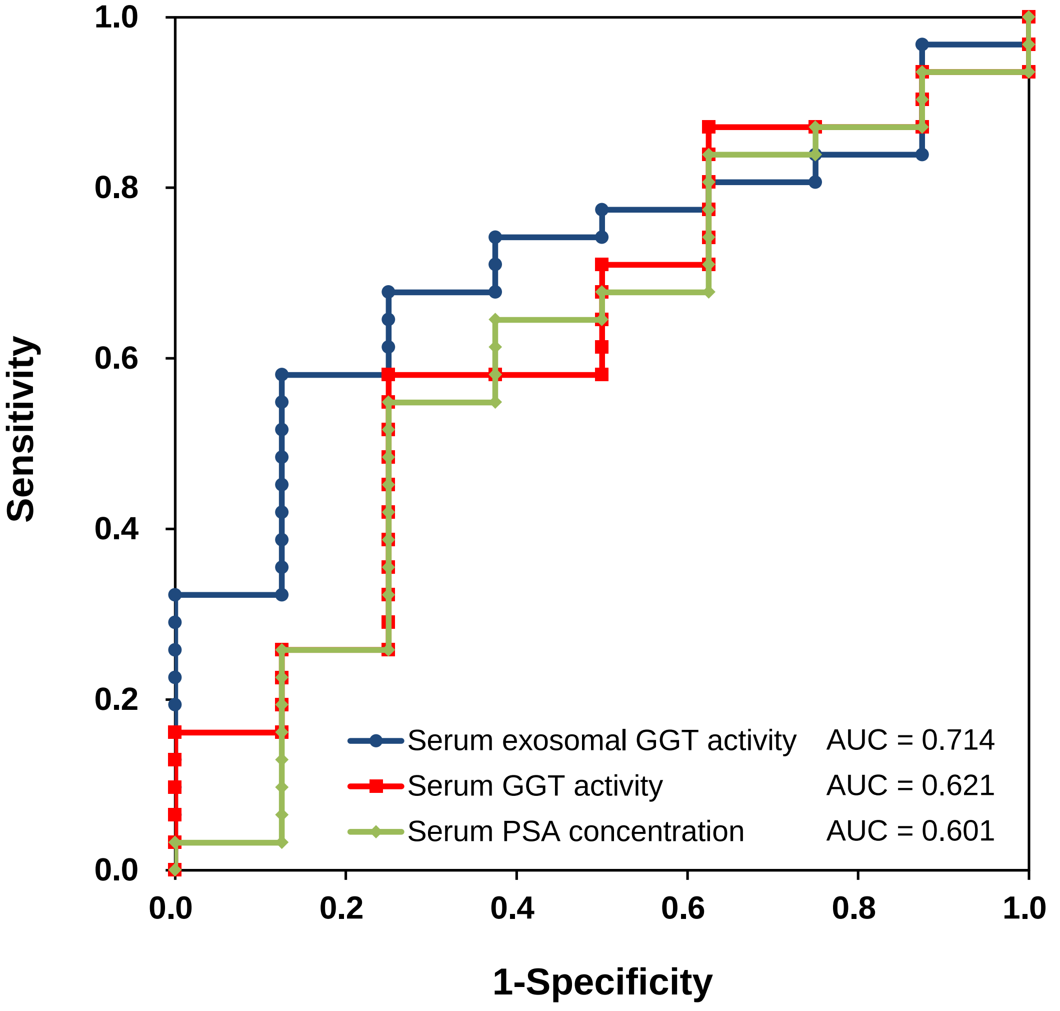

Figure S2. ROC curve analysis of PC and BPH patients.
The ROC curve was plotted and AUC was calculated for serum exosomal GGT activity, serum GGT activity and serum PSA concentration. The data for the sensitivity and 1 minus the specificity were plotted on a continuous scale.
